# Catalpol alleviates amyloid- generation and neuronal oxidative stress injury via activating the Keap1-Nrf2/ARE signaling pathway in the immortalized lymphocytes from patients with late-onset Alzheimer’s disease and SKNMC cells co-culture model

**DOI:** 10.22038/ijbms.2024.78543.16982

**Published:** 2024

**Authors:** Caixia Xiang, Yunwei Lu, Renjuan Hao, Yuyan Wei, Yingchao Hu, Guran Yu

**Affiliations:** 1 Department of Neurology, Jiangsu Province Hospital of Chinese Medicine, the Affiliated Hospital of Nanjing University of Chinese Medicine, Nanjing, Jiangsu Province, China; 2 Cent Hosp Enshi Tujia & Miao Autonomous Prefectur, 158 Wuyang Ave, Enshi 445000, Hubei, Peoples R China; 3 Department of Neurology, Shenzhen Traditional Chinese Medicine Hospital, China; # These authors contributed equally to this work

**Keywords:** Alzheimer’s Disease Amyloid beta-Peptides Apoptosis, Catalpol, Oxidative stress

## Abstract

**Objective(s):**

To assess the effect of catalpol, the major bioactive constituents of *Rehmannia glutinosa*, on our Alzheimer’s disease (AD) *in vitro* model.

**Materials and Methods:**

We employed the immortalized lymphocytes (lymphoblastoid cell line, LCL) from late-onset AD patients and co-cultured “them” to mimic the pathological process of late-onset AD and investigated the effect of catalpol on our AD *in vitro* model.

**Results:**

In the co-culture model, AD-derived LCL triggered excessive Aβ1-42 in SKNMC cells due to its high levels of oxidative stress and resulted in neuronal oxidative stress injury through inhibiting Keap1-Nrf2/ARE signaling pathway. Treatment with catalpol and N-acetylcysteine (NAC), an antioxidant, prevented the AD LCL-induced Aβ1-42 overproduction and reduced the level of β-site amyloid precursor protein cleaving enzyme-1 (BACE1) and amyloid precursor protein (APP)-C99. Catalpol and NAC also enhanced the antioxidant capacity and reduced apoptosis in SKNMC cells co-cultured with AD LCL. The anti-oxidative effect of catalpol was antagonized by ML385, the Nrf2 inhibitor. Therefore, we speculate that the antioxidant and anti-apoptotic effects of catalpol are mediated by activating the Keap1-Nrf2/ARE signaling pathway.

**Conclusion:**

Catalpol affects the anti-Aβ generation and the antioxidative and antiapoptotic properties in the AD co-cultured model. So, it might be a novel natural drug and offer a potential therapeutic approach for AD.

## Introduction

Alzheimer’s disease (AD) is the most common and incurable neurodegenerative disease affecting millions of individuals worldwide. There are two forms of AD: sporadic or late-onset AD and familial type. The sporadic or late-onset one develops in 95-98% of cases, while the familial type is limited to 2-5% (1-5). Mounting evidence shows that late-onset AD is a heterogeneous and systemic disease in which peripheral lymphocytes, inflammation, and oxidative stress play roles (6, 7). Therefore, currently employed cell models cannot entirely simulate late-onset AD *in vitro*.

Compared with age-matched control subjects, AD patients have shown higher levels of oxidative stress, especially in late-onset AD ones (8-10). Peripheral lymphocytes in AD patients also showed higher levels of oxidative markers (11-13). A large body of evidence determines that oxidative stress could lead to Aβ generation via various pathways. Oxidative stress contributes to the overexpression of BACE1 by enhancing activation of both the hypoxia-inducible factor-1alpha (HIF-1αdd) and the MAP kinase c-Jun N-terminal kinase (JNK), as well as opposing cGMP production, and ultimately results in the overproduction of Aβ (14-17). A previous study has reported that peripheral B lymphocytes from late-onset AD can induce Aβ plaque formation in SKNMC cells (6). Collectively, we propose that peripheral B lymphocytes from late-onset AD are associated with oxidative stress. Accordingly, we aimed to use immortalized B lymphocytes (lymphoblastoid cell line, LCL) obtained from late-onset AD patients and co-cultured them with SKNMC cells to investigate the curbing effects of catalpol on Aβ generation and neuronal antioxidant stress injury effects of catalpol.

Catalpol, an iridoid glycoside, is extracted from *Rehmannia glutinousa, *which plays a significant role in preventing and treating late-onset AD in traditional Chinese medicine (18, 19). Catalpol has been reported to have various neuroprotective properties, such as inhibiting Aβ generation, antioxidant, anti-apoptotic, anti-aging, anti-inflammatory, etc (20-27). In particular, catalpol has been proven to have prophylactic and therapeutic effects on AD both *in vitro* and *in vivo* (24, 28). 

In the present study, we explored the effects of catalpol on the SKNMC cells, which previously have been demonstrated to have the capability to form Aβ (6, 29), co-cultured with late-onset AD LCL. We investigated the changes in the Aβ generation, oxidant stress, and neuronal apoptosis. In addition, the role of Kelch-like ECH-associating protein 1- nuclear factor erythroid 2 related factor 2/ antioxidant response element (Keap1-Nrf2/ARE) signaling pathway activation on the antioxidant stress in SKNMCs was explored in depth to determine the underlying mechanism of catalpol in preventing the pathogenesis and progress of late-onset AD (30). 

## Materials and Methods

***Peripheral B lymphocyte isolation and immortalization***

Venous blood was taken from one late-onset AD patient (Mini-Mental State Examination score=15) in Jiangsu Province Hospital of Chinese Medicine (Jiangsu, China): one age and sex-matched healthy donor. The anticoagulant venous blood was carefully dropped on the surface of the Lymphocyte Separation Medium (Human)(TBD, LTS1077, China), and peripheral blood mononuclear cells (PBMCs) were isolated. The B95-8 cell line and its culture medium were frozen and thawed three times and then filtered through a 0.22 μm filter to get a liquid containing Epstein-Barr virus(EBV). EBV, which entered B lymphocytes by CD21 surface receptors, was added to the PBMCs to transform B lymphocytes to obtain permanent B lymphocytes *in vitro* (6, 29). After that, cyclosporine A (2 μg/ml) was added to deactivate cytotoxic T lymphocytes(6). Lymphocytes were seeded in one 24-well plate and incubated for three weeks in a 37 ^°^C, humidified atmosphere containing a 5% carbon dioxide incubator. The culture medium was changed every 5-7 days. Thereafter, the clustered-growing LCL cells were discovered. During this period, the culture medium was changed every other day. Subsequently, the LCL cells were transferred to T25 flasks and subcultured every three days.

Implementing all experiments involving human participants was in accordance with the ethical standards of the Ethics Committee of Jiangsu Hospital of Traditional Chinese Medicine (2020NL-102-03, 2020NL-102-05).

***Cell culture***

LCL cells were cultured in Roswell Park Memorial Institute RPMI-1640 medium (GIBCO, USA) containing 20% fetal bovine serum (FBS) (GIBCO, USA), penicillin (100 U/ml), streptomycin (100 mg/ml), and amphotericin B (100 mg/ml) in a 37 ^°^C, humidified atmosphere containing 5% carbon dioxide incubator. The LCL cells grew in suspension in T25 flasks with 10 ml of culture medium. The culture medium was routinely changed every two days by removing 5 ml of culture medium above the cells and replacing it with an equal volume of fresh medium. 

SKNMC cells were obtained from Fu Heng Biology Co. Ltd (Shanghai, China) and cultured in Minimum Essential Medium (MEM) (GIBCO, USA) containing 10% FBS (GIBCO, USA), 100 mg/ml amphotericin, 100 mg/ml streptomycin, and 100 U/ml penicillin at 37 ^°^C in a 5% CO2/95% air incubator. The culture medium was replaced every two days.

***Co-culture of LCL cells /SKNMC cells model in vitro***

LCL cells were cultured on transwell inserts (1 μm or 0.4 μm pore size, Corning Costar) in the 6-well or 24-well plate above the SKNMC cells layer.

***Drugs and treatment***

Catalpol powder (purity≥98%) was purchased from Chengdu Must Bio-technology Co. Ltd (Sichuan, China) and dissolved in phosphate-buffered saline (PBS, PH 7.4) (GIBCO, USA) to a concentration of 8 mmol/L. Then, the aliquots were stored in the refrigerator at -20 ^°^C. Unless otherwise stipulated, LCL cells and SKNMCs were co-cultured for 24 hr. Thereafter, the co-culture model was treated with or without catalpol or N-acetyl-L-cysteine (NAC) (Beyotime, China) as the positive control or Nrf2 inhibitor ML385 (Med Chem Express, USA). Twenty-four hours later, SKNMCs were harvested for further analysis. 

***3-(4,5-dimethylthiazol-2-yl)-2,5-diphenyltetrazolium bromide (MTT) assay***

The viability of SKNMC cells incubated with catalpol (0-200 μM) for 24 hr was measured by MTT (Sigma, USA) assay. Briefly, SKNMCs were planked with a density of 5×10^3^cells per well and then incubated for 24 hr. Following catalpol treatment for 24 hr, 20 μl of MTT (5 mg/ml) was added to the cells and incubated for four hours. The medium was discarded, and 150 μl dimethyl sulfoxide (DMSO) (Sigma, USA) was added to each well. After the samples were shaken for 10 min on the microporous quick shaker, formazan products were formed in the viable SKNMCs. Finally, the light absorbance of each well was measured using a microplate reader (BIOTEK, ELX800, USA) at a wavelength of 490 nm.

The effect of catalpol on the viability of SKNMC cells co-cultured with AD LCL cells was measured using an MTT assay. SKNMC cells were co-cultured with LCL cells in 24-well plates. After 24 hr, the co-culture model was treated with catalpol (0–150 μM). Then, 60 μl of MTT (5 mg/ml) was added into each well and incubated at 37 ^°^C. The medium was discarded four hours later, and 500 μl DMSO was added to each well. After shaking the plates for 10 min on the microporous quick shaker, the formazan was dissolved in DMSO. Finally, the absorbance of each well was measured at a wavelength of 490 nm.

***Cell counting Kit-8 (CCK-8) assay***

The viability of AD LCL cells incubated with catalpol (0–200 μM) for 24 hr was determined using CCK-8 Kit (Sigma, USA) assay. AD LCL cells were seeded in 96-well plates (1×10^4 ^cells per well) and incubated for 24 hr. Following catalpol treatment for 24 hr, 10 μl of CCK-8 was added to each well and incubated for two hours at 37 ^°^C. Finally, the light absorbance of each well was measured at 450 nm.

***Immunofluorescence staining***

SKNMCs co-cultured with LCL cells were cultivated on coverslips in 24-well plates and incubated for 24 hr. After treating with catalpol (10, 50, 100 μM) or ML385 (10 μM) for 24 hr, SKNMCs were washed with ice-cold PBS twice and fixed with 4% paraformaldehyde at room temperature for 10 min. Then, cells were permeabilized with 0.3% Triton X-100 at room temperature for 30 min followed by being blocked in immunol staining blocking buffer (Beyotime, China) and incubated with primary antibodies targeting the receptors expressed on Aβ1-42, Nrf2 at 4 ^°^C overnight (Table 1). Thereafter, the cells were exposed to fluorescent-labeled secondary antibodies (1:1000, Proteintech, USA) for one hour at room temperature in the dark. The nuclei of SKNMCs were stained with 2-(4-amidinophenyl)-6-indolecarbamidine dihydrochloride (DAPI) for 10 min, and cells were observed and photographed by using a fluorescence microscope (DS-Qi2, NIKON, Japan). Finally, each image was analyzed by Image J.

*Western blot analysis*

SKNMC cells were co-cultured with LCL cells in 6-well plates and then incubated for 24 hr at 37 ^°^C, followed by being treated with catalpol (10, 50, 100 μM), NAC (1 mM), or ML385 (10 μM) for 24 hr. After removing transwell inserts and washing cells with ice-cold PBS three times, SKNMCs were lysed in Radioimmunoprecipitation assay buffer (RIPA)(Beyotime, China) containing 1% protease inhibitor cocktails. Twenty minutes later, lysates were harvested and centrifuged. A nuclear and cytoplasmic protein extraction kit (Beyotime, China) was then used to extract the nuclear/cytoplasmic proteins according to the standard protocol, and the concentration of protein was determined by BCA protein assay kit (Beyotime, China). 200 μl BCA reagent (Reagent A: Reagent B=50:1) was added to each sample. After 30 min of incubation at 37 ^°^C, each sample was detected by light absorbance at 540 nm with a microplate reader, and then the protein concentration was determined. Equal amounts (10-20 μg) of protein were separated by 10% sodium dodecyl sulfate-polyacrylamide gel electrophoresis (SDS-PAGE) gel for 120 min (Voltage:90v). Then they were transferred to polyvinylidene fluoride (PVDF) membranes. Membranes were then blocked in 5% non-fat milk in tris-buffered saline with 0.1% Tween 20 (TBST, pH 7.6) and coated by the appropriate primary antibodies (Table 1), followed by incubation overnight at 4 ^°^C. After one hour of incubation with secondary antibodies (1:3000, Servicebio, China) at room temperature, the membranes were washed three times with TBST for 10 min each time, and enhanced chemiluminescence (Biosharp, China) was used to make western blots visible in the ChemiDoc XRS+Gel Imaging System (BIO-RED, USA)(31). Finally, band density was semi-quantitatively analyzed with Image Lab software.

***Reactive oxygen species (ROS) generation***

The intracellular ROS levels were quantitatively and qualitatively detected by a 2’,7’-Dichlorodihydrofluorescein diacetate (DCFH-DA) fluorescence probe (Beyotime, China). SKNMCs were cultured on coverslips in 24-well plates and then co-cultured with LCL cells, followed by incubation for 24 hr at 37 ^°^C. Thereafter, the cells were treated with catalpol (10, 50, 100 μM), NAC (1 mM), or ML385 (10 μM) and incubated for 24 hr. SKNMCs were then incubated with a DCFH-DA fluorescence probe for 30 min in a 37 ^°^C, 5% CO2/95% air incubator in the dark. After being labeled with DCFH-DA, cells were detected with fluorescence microscopy, and fluorescent images were measured using Image J.

*Malondialdehyde (MDA) level assay*

The quantification of MDA was determined using a Lipid Peroxidation MDA Assay Kit (Beyotime, China)(32). SKNMCs co-cultured with LCL cells in 6-well plates were incubated for 24 hr, and cells were treated with catalpol (10, 50, 100 μM), NAC (1 mM), or ML385 (10 μM). After 24 hr of incubation, transwell inserts were removed, and the medium was discarded. SKNMCs were then washed with ice-cold PBS and lysed in RIPA. Thereafter, lysates were harvested and centrifuged, and the supernatant was extracted. The protein concentration of the supernatant was then measured by a BCA protein assay kit, and MDA detection working fluid was added to each sample, then heated at 100 ^°^C for 15 min. Subsequently, the samples were cooled to room temperature in a water bath and centrifuged. The supernatant was extracted and detected by light absorbance at 532 nm with a microplate reader.

***Determination of reduced glutathione (GSH) and oxidized glutathione (GSSG) levels***

SKNMCs were co-cultured with LCL cells in 6-well plates and incubated for 24 hr in a 37 ^°^C, 5% CO_2_/95% air incubator. After 24 hr of treatment with catalpol (10, 50, 100 μM), NAC (1 mM), or ML385 (10 μM), the transwell inserts were taken away, and the medium was discarded. SKNMCs were harvested and washed with ice-cold PBS once for the subsequent measurement. Each sample’s GSH and GSSG content were estimated by a GSH and GSSG Assay Kit (Beyotime, China). The specific assay process of GSH/GSSG followed the manufacturer’s protocol. Finally, the samples were added to a 96-well plate and measured at 412 nm with a microplate reader. 

***Catalase (CAT) activity***

CAT activity was measured using the Catalase Assay Kit (Beyotime, China) according to the manufacturer’s instructions. Briefly, SKNMCs in co-culture with LCL cells were incubated for 24 hr in 6-well plates. The cells were then treated with catalpol (10, 50, 100 μM), NAC (1 mM), or ML385 (10 μM). 24 hr later, SKNMCs were washed with ice-cold PBS three times, and RIPA was added to the lyse cells. The lysates were diluted with catalase detection buffer, and hydrogen peroxide solution was added to each sample. Following the reaction at 25 ^°^C for 5 min, catalase reaction termination solution was added, and the chromogenic working solution was added within 15 min. The samples were analyzed by measuring the absorbance at 520 nm using a microplate reader.

***Measurement of superoxide dismutase (SOD) activity***

The activity of SOD was measured by Cu/Zn-SOD and Mn-SOD Assay Kit with WST-8 (Beyotime, China)(33). WST-8 reaction with superoxide anion is catalyzed by xanthine oxidase to produce water-soluble formazan dye, and SOD could inhibit the reaction. The enzyme activity of SOD was then calculated by colorimetric analysis of the WST-8 product. Briefly, SKNMCs were routinely co-cultured with LCL cells in 6-well plates and incubated for 24 hr. After drug treatment for 24 hr, SKNMCs were homogenized and centrifuged, and the supernatant was collected for the subsequent measurement. The specific operation procedure was strictly carried out according to the manufacturer’s instructions. Finally, the absorption at a wavelength of 450 nm was detected using a microplate reader.

***Flow cytometric analysis of apoptosis***

Annexin V-fluorescein Isothiocyanate (FITC)/Propidium Iodide (PI) Apoptosis Detection Kit (Vazyme, China) was used to assess the apoptotic activity in SKNMCs co-cultured with LCL cells. SKNMCs were co-cultured with LCL cells in 6-well plates. After 24 hr of incubation, the cells were treated with catalpol (10, 50, 100 μM), NAC (1 mM), or ML385 (10 μM). 24 hr later, SKNMCs were harvested and washed with PBS. Thereafter, cells were resuspended in 100 μl 1×binding buffer, and 5 μl of V-FITC and 5 μl of PI were added into each sample respectively, followed by being incubated for 10 min at room temperature in the dark. Then, 400 μl of 1×binding buffer was added to each sample, and apoptotic activities of cells were examined by using flow cytometry (FACS Celesta, BD Biosciences) within one hour. Finally, the data was analyzed with Flow Jo.

***Hoechst33258 staining for apoptosis analysis***

SKNMC cells were seeded on coverslips in 24-well plates and then co-cultured with LCL cells. After 24 hr of incubation, the cells were treated with catalpol (10, 50, 100 μM), NAC (1 mM), or ML385 (10 μM) and incubated for 24 hr. SKNMCs were washed twice with PBS and fixed with 4% paraformaldehyde at room temperature for 20 min. Thereafter, SKNMCs were washed with PBS 3 times and then were incubated with the Hoechst 33258 (Beyotime, China) stain solution for 30 min at room temperature for subsequent imaging using a fluorescence microscope and analyzing with Image J.

***5,5*** ***′*** ***6,6*** ***′*** ***-tetrachloro-1,1*** ***′*** ***,3,3*** ***′*** ***-tetraethyl-imidacarbocyanine iodide (JC-1) mitochondrial membrane potential assay***

SKNMC cells were cultivated on coverslips in 24-well plates and then co-cultured with LCL cells, followed by incubation for 24 hr. SKNMCs were then treated with catalpol (10, 50, 100 μM), NAC (1 mM), or ML385 (10 μM). After 24 hr of incubation, SKNMCs were washed with PBS once and labeled with JC-1 dye (Beyotime, China)(34). Then, the cells were incubated for 20 min at 37 ^°^C. Finally, a fluorescence microscope was used for cell imaging, and images were measured by Image J.

***Statistical analysis***

All data were statistically analyzed using IBM SPSS Statistics 26.0 software (SPSS Inc, USA). Data are presented as mean±standard error and followed by the normal distribution test. One-way ANOVA was utilized to compare the experimental results followed by an appropriate individual *post hoc* test (Dunnett’s T3 or Tukey’s test). A threshold of *P*<0.05 was utilized for statistical significance. All experiments were replicated three times independently.

## Results

***Viability of SKNMC cells treated with different concentrations of catalpol***

**Figure 1:**
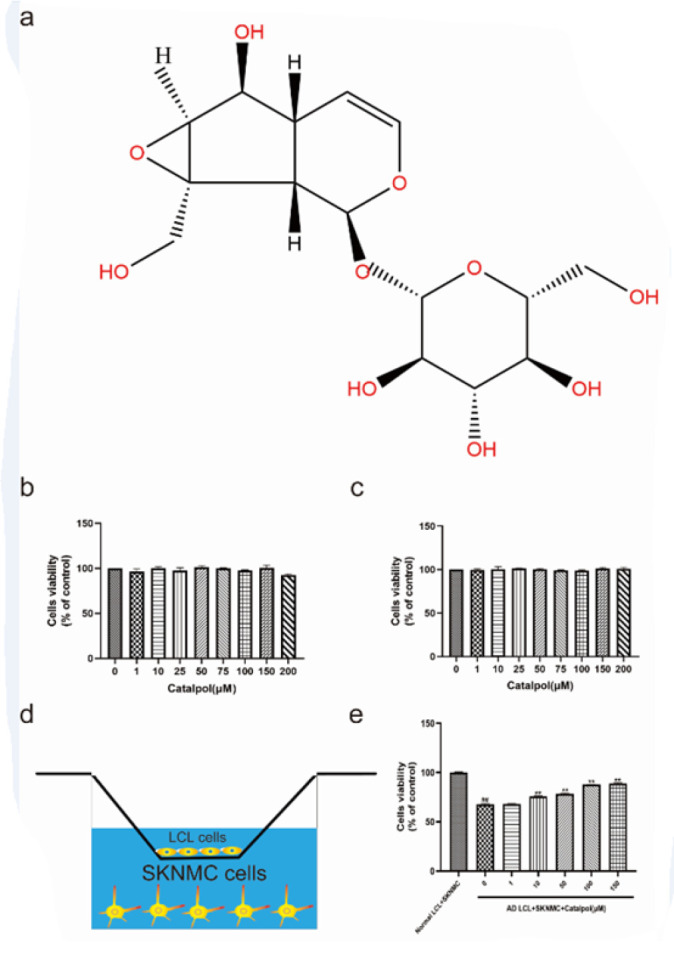
(a) Chemical structure of catalpol. (b) Effect of catalpol on the cell viability of SKNMCs. the viability of SKNMCs treated with different concentrations of catalpol (0,1, 10, 25, 50, 75, 100, 150, and 200 μM) was measured by MTT assay. The viability of SKNMCs with catalpol treatment had no statistical difference compared to the control (*P>*0.05). (c) The viability of AD LCL cells was measured by CCK8 assay. With and without catalpol treatment, there was no significant difference in the activity of AD LCL cells (*P>*0.05). (d) Schematic diagram of SKNMCs/LCL cells co-culture. (e) Viability of SKNMCs in co-culture with LCL cells treated with catalpol (0,1,10, 50, 100, and 150 μM) was measured by MTT assay

Before probing the effects of catalpol on SKNMC cells co-cultured with AD LCL cells, we examined the effect of catalpol on the activity of SKNMCs by MTT assay. SKNMCs were incubated with different concentrations of catalpol (1, 10, 25, 50, 75, 100, 150, and 200 μM) for 24 hr. Cell activity was not significantly different between each catalpol treatment group and the control (Figure 1b). The results indicated that the experimental concentrations of catalpol had no significant effect on the activity of SKNMCs.

***Viability of AD LCL cells treated with different concentrations of catalpol***

To assess the toxicity of catalpol on AD LCL cells, we detected the effect of catalpol on the viability of AD LCL cells by the CCK-8 assay. AD LCL cells were treated with different concentrations of catalpol (1, 10, 25, 50, 75, 100, 150, and 200 μM) for 24 hr. There was no significant difference in cell viability between each catalpol treatment group and the control (Figure 1c). This indicated that the experimental concentrations of catalpol had no significant effect on the viability of AD LCL cells.

***Viability of SKNMC cells co-cultured with AD LCL cells treated with different concentrations of catalpol***

To explore optimal neuroprotective concentration of catalpol against AD LCL cells induced cytotoxicity, the co-cultured models were treated with different concentrations of catalpol (1, 10, 50, 100, 150 μM) for 24 hr. The MTT assay showed that the viability of SKNMCs co-cultured with AD LCL cells was significantly reduced compared to the control. Catalpol dose-dependently prevented cytotoxicity of AD LCL to SKNMCs at 10-100 μM. In addition, there was no significant difference in cell viability between 100 μM and 150 μM (Figure 1e). 

***Catalpol prevented Aβ generation of SKNMCs in co-culture with AD LCL cells***

**Figure 2:**
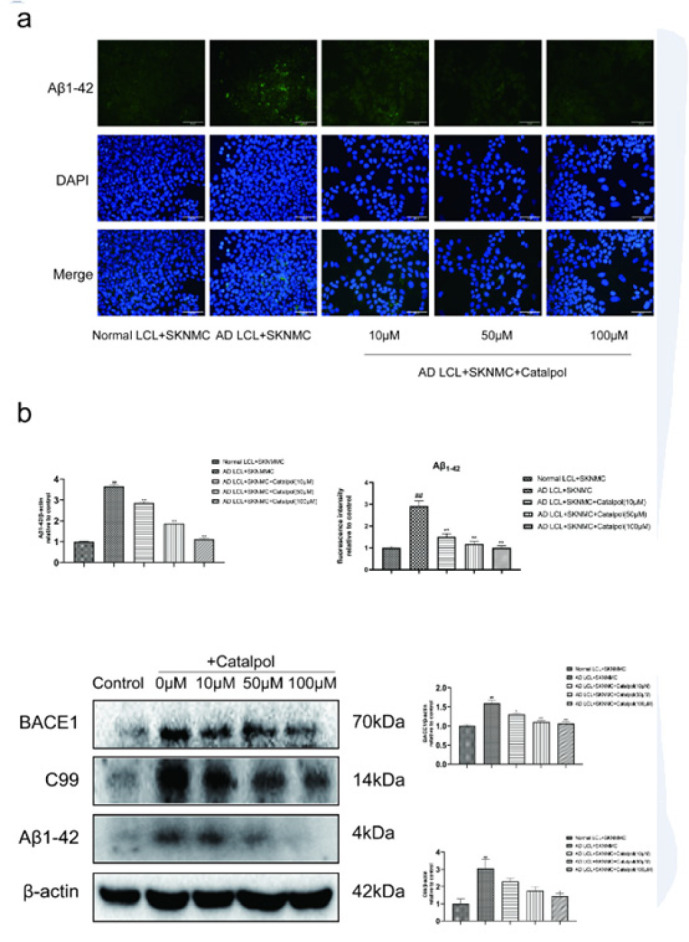
Catalpol prevented beta-amyloid generation in SKNMCs co-cultured with AD LCL cells

The production of Aβ1-42 in co-cultured SKNMCs was tested by immunofluorescence staining and western blot. Meanwhile, the expression of Aβ generation-related proteins β Amyloid precursor protein lyase 1 (BACE1) and C99 (C-terminal fragment-β, β-CTF) were detected by western blot. Compared with the control, the expression of Aβ1-42, BACE1, and C99 in SKNMC cells co-cultured with AD LCL cells was significantly increased. Catalpol reduced AD LCL cells-induced Aβ_1-42_, BACE1, and **C99 levels rise** in a dose-dependent manner from 10 to 100 μM (Figure 2a, 2b). 

***Catalpol suppressed oxidative stress expressed in SKNMCs co-cultured with AD LCL cells***

**Figure 3:**
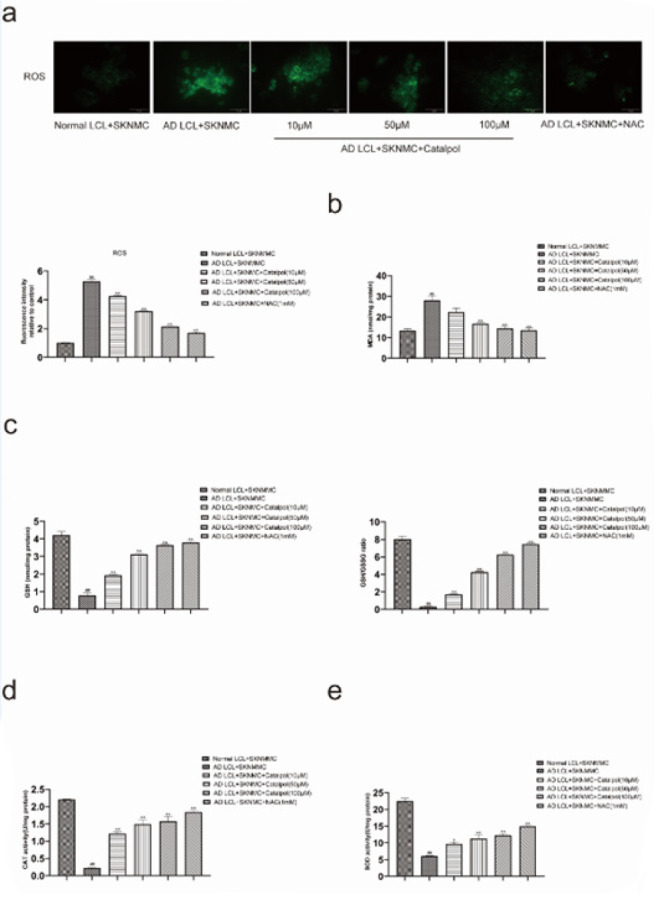
Protective potential of catalpol against oxidative stress in SKNMCs co-cultured with AD LCL cells

SKNMC cells were co-cultured with AD LCL cells for 24 hr and subsequently treated without or with catalpol (10, 50, 100 μM) or 1 mM NAC, an antioxidant, for 24 hr. The level of ROS was determined by the DCFH-DA fluorescent probe. Stimulating SKNMC cells with AD LCL cells, the level of ROS was significantly increased in co-cultured SKNMCs. The increase was reversed by treatment with catalpol and NAC (Figure 3a).

Thereafter, we detected the levels of MDA, GSH, GSH/GSSG, CAT, and SOD by respective assay kits. Stimulating SKNMC cells with AD LCL cells resulted in markedly increased levels of MDA and decreased activities of antioxidant enzymes (GSH, GSH/GSSG, CAT, and SOD). Like NAC, catalpol reversed the corresponding changes in a dose-dependent manner from 10 to 100 μM (Figure 3b, 3c, 3d, 3e).

***Catalpol inhibited the activation of the AD LCL cells-triggered apoptotic pathway in SKNMC cells***

**Figure 4:**
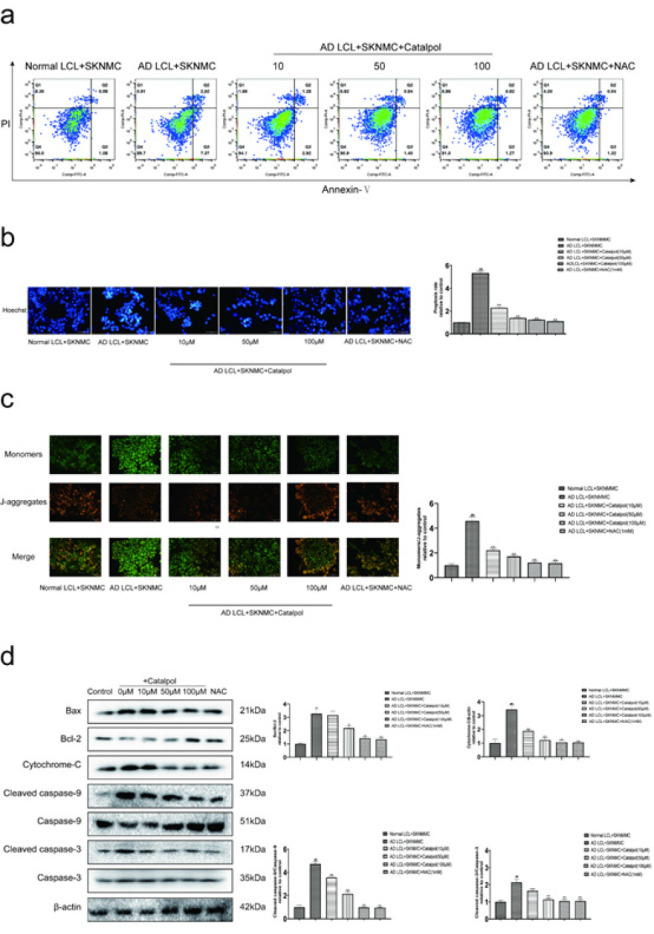
Catalpol inhibited apoptosis of SKNMCs co-cultured with AD LCL cells

The apoptosis of SKNMC cells was evaluated by flow cytometric analysis, Hoechst 33258 staining, and JC-1 staining, while the expressions of apoptosis-related proteins were determined by western blot. Detected by flow cytometric analysis, treatment with catalpol (10, 50, 100 μM) and NAC decreased the apoptosis rates of SKNMC cells in AD LCL cells/SKNMC cells co-cultures (Figure 4a). Hoechst 33258 staining also showed more Hoechst-positive cells emerged in AD groups; catalpol and NAC reduced the quantity of AD LCL cell-induced Hoechst-positive cells (Figure 4b). JC-1 staining indicated that mitochondrial membrane potential was decreased in SKNMC cells after stimulation with AD LCL cells. Treatment with catalpol and NAC reversed the disruption of mitochondrial membrane potential in co-cultured SKNMC cells (Figure 4c). The levels of Bax/Bcl-2, cytochrome-C, cleaved caspase-9/caspase-9, and cleaved caspase-3/caspase-3 were markedly increased in SKNMC cells co-cultured with AD LCL cells. The increase was reversed by treatment with catalpol and NAC (Figure 4d). 

*Effects of catalpol on the expression of the Nrf2 pathway proteins and nuclear translocation of Nrf2 in AD LCL cells-stimulated SKNMC cells*

**Figure 5:**
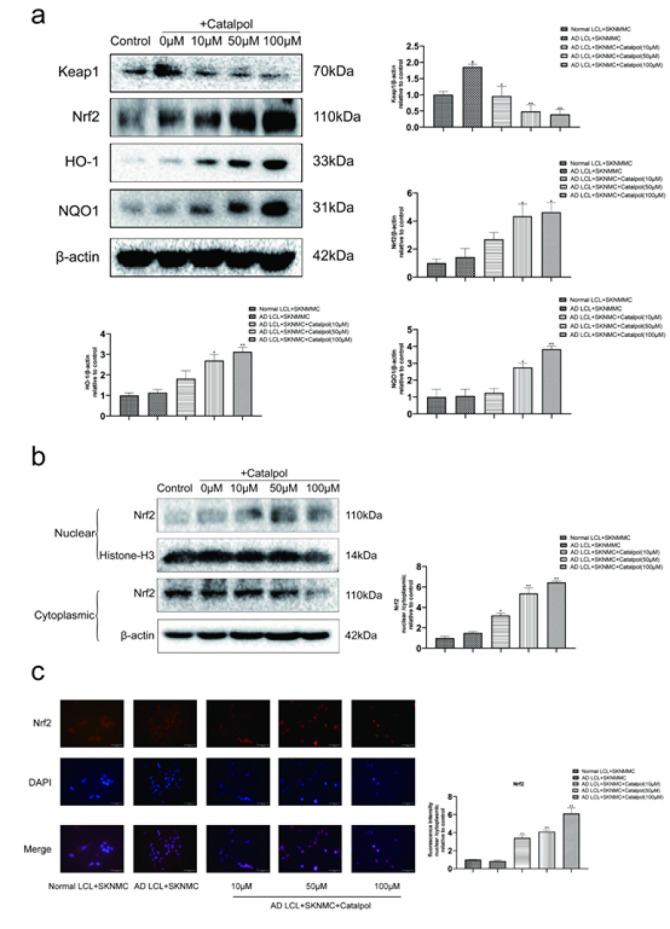
Effects of catalpol on Keap1/Nrf2 signaling pathway

Western blot analysis showed that the AD group showed lower levels of Nrf2, heme oxygenase-1 (HO-1), and quinone oxidoreductase-1 (NQO1) and higher expression of Keap1 in SKNMC cells. Catalpol dose-dependently increased the expressions of Nrf2, HO-1, and NQO1 and reduced the level of Keap1 at 10-100 μM (Figure 5a).

Meanwhile, Nrf2 nuclear translocation was detected by western blot and immunofluorescent staining. Co-cultured with AD LCL cells, Nrf2 nuclear translocation in SKNMC cells was significantly inhibited. Nrf2 expression was significantly decreased in cytoplasm while being elevated in the nuclear fraction in AD LCL cells-stimulated SKNMC cells treated with catalpol. This demonstrated that catalpol could promote nuclear translocation of Nrf2 in AD LCL cells-stimulated SKNMC cells in a dose-dependent manner from 10 to 100 μM (Figure 5b, 5c).

***Catalpol treatment decreased oxidative stress in SKNMCs co-cultured with AD LCL cells by activating the Keap1-Nrf2/ARE signaling pathway***

**Figure 6:**
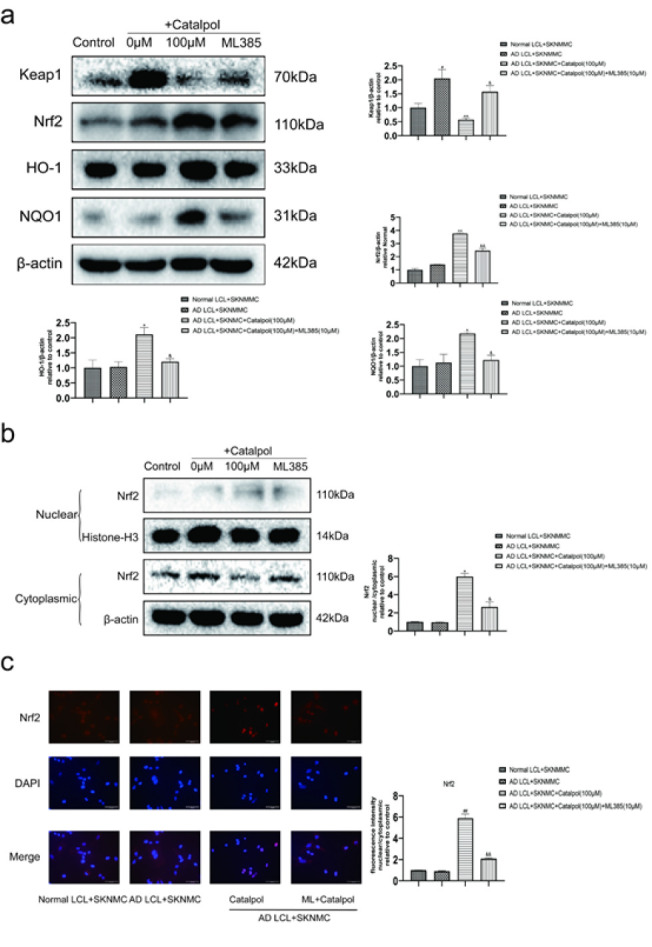
ML385 blocked the effects of catalpol on the expressions of proteins associated with the Keap1/Nrf2 signaling pathway and Nrf2 nuclear translocation

The co-cultures were treated with or without catalpol (100 μM) or Nrf2 inhibitor ML385 (10 μM) for 24 hr. ML385 decreased the expressions of Nrf2, HO-1, and NQO1, elevated the level of Keap1 and blocked Nrf2 nuclear translocation of SKNMC cells in catalpol-treated co-cultures. These results showed that ML385 could inhibit the effect of activating Keap1-Nrf2/ARE signaling pathway in catalpol (Figure 6a, 6b, 6c).

**Figure 7:**
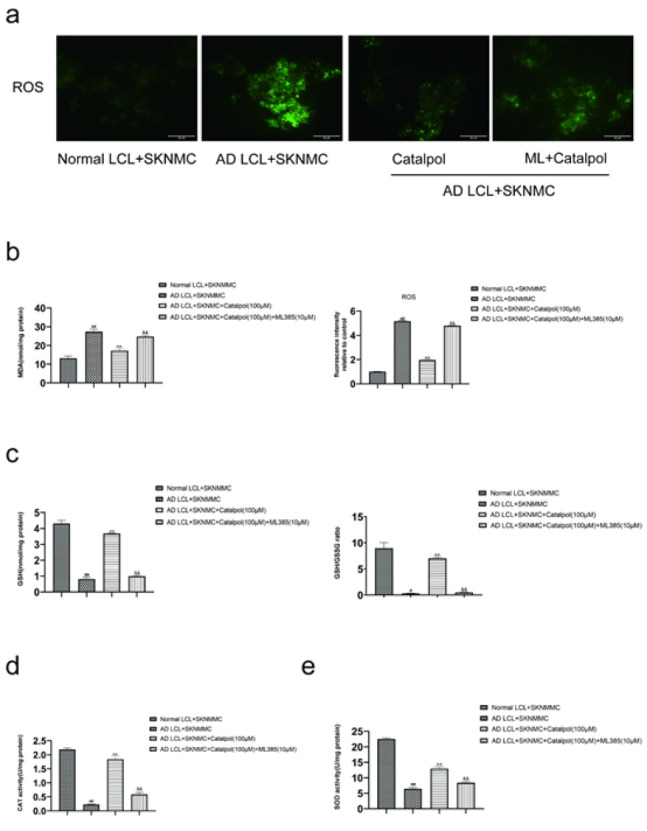
Effect of ML385 on the levels of oxidative stress markers in the catalpol treatment group

**Figure 8:**
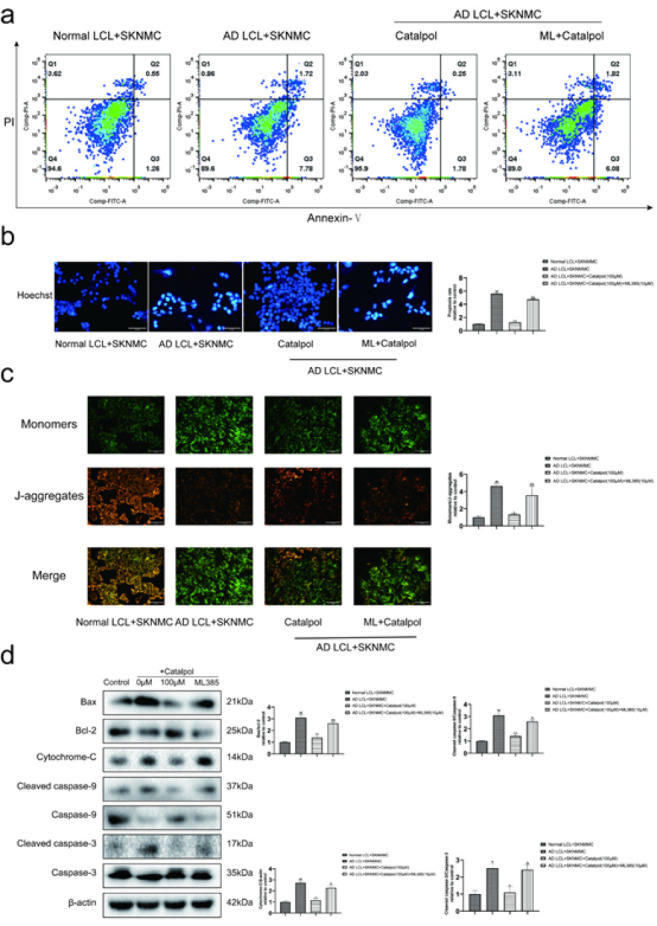
ML385 attenuated the anti-apoptotic effect of catalpol on SKNMC cells co-cultured with AD LCL cells

Compared with the group treated by catalpol, ML385 apparently reversed catalpol-induced decreased levels of ROS and MDA, increased activities of GSH, CAT, and SOD, and up-regulated the ratio of GSH/GSSG (Figure 7a, 7b, 7c, 7d, 7e). Meanwhile, ML385 alleviated the effect of catalpol in reducing apoptosis rate, recovering the decline of mitochondrial membrane potential, and down-regulating the expressions of apoptosis-related proteins in SKNMC cells co-cultured with AD LCL cells (Figure 8a, 8b, 8c, 8d).

**Table 1:** List of the antibodies used in Western blot analysis

Antibody	Host	Dilution	Source (reference)
APP C-terminal	Rabbit	1:2000	Serotec (158578)
β-Amyloid (1-42)	Rabbit	1:1000	ChinaPeptides (#14974)
BACE-1	Rabbit	1:1000	AiFang biological (AF301306)
Caspase9	Mouse	1:1000	Cell Signaling Technology (#9508)
Caspase3/p17/p19	Mouse	1:1000	Proteintech (66470-2-Ig)
BAX	Rabbit	1:1000	Proteintech (50599-2-Ig)
Bcl2	Rabbit	1:1000	Proteintech (12789-1-AP)
Cytochrome C	Rabbit	1:5000	Cell Signaling Technology (#11940)
Nrf2	Rabbit	1:2000	Proteintech (16396-1-AP)
Keap1	Rabbit	1:1000	AiFang biological (AF06660)
NQO1	Rabbit	1:2000	AiFang biological (AF301136)
HO-1/HMOX1	Rabbit	1:2000	Proteintech (10701-1-AP)
Beta Actin	Mouse	1:5000	Proteintech (66009-1-Ig)
Histone-H3	Rabbit	1:1000	Proteintech (17168-1-AP)

(a) ROS generation in SKNMCs co-cultured with LCL cells exposed to different concentrations of catalpol, NAC (1mM) for 24 hr. Fluorescence of intracellular ROS generation was grabbed under a fluorescence microscope at a magnification of ×400. The fluorescence intensity was semi-quantified using Image J. (b) The level of MDA was measured. (c) Content of GSH and the ratio of GSH/GSSG were measured. (d) Level of catalase activity was measured. (e) Relative enzyme activities of SOD were measured. All data are presented as the mean±SD of three separate experiments performed in triplicate. #*P<*0.05, ^##^*P<*0.01 versus control (SKNMCs co-cultured with normal LCL cells). *P<0.05, **P<0.01 versus the AD group (SKNMCs co-cultured with AD LCL cells)

(a) Effect of catalpol against apoptosis in SKNMCs co-cultured with AD LCL cells was detected by flow cytometric analysis. Cells were stained with V- FITC/ PI. The data was analyzed using Flow Jo. (b) All nuclei were stained by Hoechst 33258 with blue fluorescence. Apoptotic nuclei stained by Hoechst 33258 presented bright blue fluorescence. Normal nuclei stained by Hoechst 33258 presented dark blue fluorescence. Images were obtained by a fluorescence microscope at a magnification of × 400. (c) Mitochondrial potential in SKNMCs in co-culture with AD LCL cells was observed by JC-1 staining. When the mitochondrial membrane potential was high, the red fluorescence represented the “J-aggregates” form. On the contrary, it displayed green fluorescence, representing the monomeric form of JC-1. (d) Catalpol suppressed the mitochondrial apoptotic signaling pathway protein expression in SKNMCs co-cultured with AD LCL cells. Western blot analysis showed the levels of Bax/Bcl-2, cytochrome c, cleaved caspase-9/caspase-9, and cleaved caspase-3/caspase-3 proteins in SKNMCs co-cultured with LCL cells with different treatments. Actin levels were measured for the confirmation of equal amount of protein loading. Images were gained by a fluorescence microscope at a magnification of × 400. The fluorescence intensity was analyzed by Image J. The data were analyzed by the ratio of green to red fluorescence intensity. All data are represented as mean±SEM from three independent experiments. ^#^*P<*0.05, ^##^*P<*0.01 versus control (SKNMCs co-cultured with normal LCL cells). *P<0.05, **P<0.01 versus the AD group (SKNMCs co-cultured with AD LCL cells)

(a) Catalpol increased total Nrf2 protein ‘HO-1 ‘NQO1 expressions and decreased Keap1 expression. (b) Catalpol increased nuclear Nrf2 expression in SKNMC cells co-cultured AD LCL cells. (c)Nrf2 expression in SKNMCs co-cultured with AD LCL cells exposed to different concentrations of catalpol for 24 hr. Fluorescence of Nrf2 in the nucleus was observed under a fluorescence microscope at 400x magnification. The fluorescence intensity was semi-quantified using Image J. All results are represented as mean±SEM from three independent experiments. ^#^*P<*0.05, ^##^*P<*0.01 versus control (SKNMCs co-cultured with normal LCL cells). *P<0.05, **P<0.01 versus the AD group (SKNMCs co-cultured with AD LCL cells)

(a) Nrf2 inhibitor (ML385) reversed increased total Nrf2 protein, HO-1, and NQO1 expressions and decreased Keap1 expression by catalpol (100 μM) treatment. (b) ML385 down-regulated increased nuclear Nrf2 expression in SKNMC cells co-cultured AD LCL cells by catalpol (100 μM) treatment. (c)Nrf2 expression in SKNMCs co-cultured with AD LCL cells with different treatments for 24 hr. The Fluorescence of Nrf2 in the nucleus was grabbed under a fluorescence microscope at a magnification of ×400. The fluorescence intensity was semi-quantified using Image J. All the results are represented as mean±SEM from three independent experiments. ^#^*P<*0.05, ^##^*P<*0.01 versus control (SKNMCs co-cultured with normal LCL cells). *P<0.05, **P<0.01 versus the AD group (SKNMCs co-cultured with AD LCL cells). ^&^*P<*0.05, ^&&^*P<*0.01 versus the catalpol (100 μM) treatment group

(a) Generation of ROS was measured by a DCFH-DA fluorescent probe. The fluorescence intensity of ROS in the catalpol treatment group was decreased compared with the AD group. The fluorescence intensity of ROS in the ML385 group was increased compared with the catalpol treatment group. Fluorescence of ROS was grabbed under a fluorescence microscope at a magnification of ×400. The fluorescence intensity was semi-quantified using Image J. (b) The level of MDA was measured after ML385 was added. (c) The content of GSH and the ratio of GSH/GSSG were measured after ML385 was added. (d) The level of catalase activity was measured after ML385 was added. (e)The relative enzyme activities of SOD were measured after ML385 was added. All the results are represented as mean±SEM from at least three independent experiments. ^#^*P<*0.05, ^##^*P<*0.01 versus control (SKNMCs co-cultured with normal LCL cells). *P<0.05, **P<0.01 versus the AD group (SKNMCs co-cultured with AD LCL cells). ^&^*P<*0.05, ^&&^*P<*0.01 versus the catalpol (100 μM) treatment group

(a) Apoptosis of co-cultured SKNMC cells with different treatments was detected by flow cytometric analysis. Cells were stained with V- FITC/ PI. The data was analyzed by Flow Jo. (b) All nuclei were stained by Hoechst 33258 with blue fluorescence. Apoptotic nuclei stained by Hoechst 33258 presented bright blue fluorescence. Normal nuclei stained by Hoechst 33258 presented dark blue fluorescence. Images were obtained by a fluorescence microscope at a magnification of ×400. (c) Mitochondrial potential in SKNMCs in co-culture with AD LCL cells was observed by JC-1 staining. When the mitochondrial membrane potential was high, the red fluorescence represented the “J-aggregates” form. On the contrary, it displayed green fluorescence, representing the monomeric form of JC-1. (d) Western blot analysis showed the levels of Bax/Bcl-2, cytochrome c, cleaved caspase-9/caspase-9, and cleaved caspase-3/caspase-3 proteins in SKNMCs co-cultured with LCL cells with different treatments. Actin levels were measured for the confirmation of equal amount of protein loading. Images were gained by a fluorescence microscope at a magnification of ×400. The fluorescence intensity was analyzed by Image J. All data are represented as mean±SEM from three independent experiments. #*P<*0.05, ^##^*P<*0.01 versus control (SKNMCs co-cultured with normal LCL cells). *P<0.05, **P<0.01 versus the AD group (SKNMCs co-cultured with AD LCL cells). ^&^*P<*0.05, ^&&^*P<*0.01 versus the catalpol (100 μM) treatment group

## Discussion

AD is an age-related neurodegenerative and systemic disorder. The pathogenesis and progression of late-onset AD are related to many factors, including Aβ plaque, tau hyperphosphorylation, oxidative stress, inflammatory response, and adaptive immunity (35). In adaptive immunity, peripheral B cells play a considerable role in AD progression since they up-regulate the expressions of proinflammatory cytokines and infiltrate into the brain parenchyma, triggering immunoglobulin accumulation around Aβ plaques and resulting in AD progression [21]. Nevertheless, peripheral B lymphocytes have a “light” side. They produce Aβ-specific antibodies and nonspecific immunoglobulin to improve AD [15-20]. Consequently, according to the stage of differentiation and activation status, peripheral B lymphocytes exert beneficial or deleterious functions [22]. In the late-onset AD hosts, B lymphocytes, as pathogenic cells, acquire pathogenic functions and exacerbate AD. Correspondingly, B lymphocyte depletion, as a therapeutic target, can block the progression of AD in three different murine transgenic models [21](36). The literature has numerous discussions about the relevance of inflammation and B lymphocytes. Reciprocally, the relationship between peripheral B cells from late-onset AD and oxidative stress is yet documented. Our present study explored the link of peripheral B cells from late-onset AD to Aβ generation and oxidative stress *in vitro*. Likewise, we also investigated the effect of catalpol on AD LCL cell-induced changes in an *in vitro* AD LCL cells/SKNMC cells co-culture model by analyzing Aβ generation, neuronal oxidative stress, and apoptosis. We also found that the underlying antioxidative mechanisms of catalpol might depend on the activation of the Keap1-Nrf2/ARE pathway in SKNMC cells.

A heavy accumulation of Aβ and Aβ-induced cytotoxicity contributes to the pathogenesis and progression of AD. Sequential cleavages of APP form Aβ. APP is firstly cleaved by BACE-1 to liberate C99. Then, C99 is cleaved by γ-secretase to liberate Aβ. Aβ length-variants are between 37 and 43 residues in length. And they comprise four principal forms, which are Aβ_37_, Aβ_38_, Aβ_40,_ and Aβ_42_, which are composed of approximately 15%, 10%, 60%, and 8% of Aβ length-variants in cerebrospinal fluid (CSF), respectively(37). Among the four subtypes, Aβ_42,_ the most neurotoxic species of Aβ, is bound up with senile plaque amyloid and prone to aggregate to form oligomer, and then goes a deep step to form amyloid fibrils. Ultimately, it results in the onset of AD. Furthermore, peripheral B cells of the late-onset AD initiate the generation of Aβ plaques in a foreign cell line *in vitro*(6). In this study, AD LCL cells, derived from one late-onset AD patient and transformed into immortalized lymphocytes by EB virus, induced more Aβ_1-42_ generation in co-cultured SKNMCs compared with the control. Meanwhile, AD LCL cells induced an increase in the expression levels of proteins BACE-1 and C99, which are involved in Aβ formation pathways in co-cultured SKNMCs. Whereas the increase was reversed by treatment with catalpol. Our data indicated that catalpol could inhibit the production of Aβ_1-42_ in late-onset AD LCL cell-induced SKNMC cells by suppressing β-cleavage of APP. In the future, we may further explore whether catalpol affects some signal pathways related to generating Aβ, such as the PI3K / Akt signal pathway.

Aβ is delivered to the mitochondrion by mitochondrial outer membrane complex transposase, binds to a variety of proteins, and then triggers mitochondrial dysfunction, eventually, destroying mitochondrial membrane potential and massive production of ROS. The excessive production of ROS directly or indirectly oxidizes and damages proteins, lipids, and nucleic acids (38). The content of MDA reflects the level of lipid peroxidation. MDA and ROS are markers of oxidative activity. GSH, CAT, and SOD have antioxidant activity and are hallmarks of antioxidant activity. The excessive deposition of Aβ oligomer in extracellular accelerates oxidative stress, while oxidative stress can also favor the formation of Aβ, forming a vicious cycle. Taken together, it is reasonable to speculate that peripheral B cells of late-onset AD can induce oxidative stress in neurocytes. In this study, AD LCL cells up-regulated the levels of ROS and MDA and down-regulated the enzymatic activity of GSH, CAT, and SOD in co-cultured SKNMCs. All these changes in SKNMC cells co-cultured with AD LCL cells were reversed by catalpol and NAC. These results further prove that catalpol can alleviate oxidative stress in AD LCL cells-induced SKNMC cells. 

Several studies showed that oxidative stress induces neuronal apoptosis (39-42). As late-onset AD LCL cells can induce oxidative stress in co-cultured SKNMC cells, we are inclined to believe that late-onset AD peripheral B cells may induce neuronal apoptosis. Consistent with a previous report that the onset of apoptosis in SKNMC cells co-cultured with AD LCL cells was observed* in vitro (6)*, our data of flow cytometry and Hoechst 33258 staining indicated that AD LCL cells induced apoptosis of co-cultured SKNMC cells. We further investigated the apoptotic pathway of SKNMC cells induced by AD LCL cells. Apoptotic pathways are divided into three categories: mitochondrial pathway (or intrinsic pathway), death receptor pathway (or extrinsic pathway), and endoplasmic reticulum pathway (43). Among them, mitochondrial apoptosis is a crucial and classical apoptotic pathway (44). Mitochondrial apoptosis is as follows: when cells are stimulated by apoptosis, the anti-apoptotic protein Bcl2 is inhibited by competitive binding, and Bax is released to form the Bax / Bak oligomeric complex. Then, the oligomeric complex causes the change of mitochondrial osmotic pressure and the repression of mitochondrial membrane potential. Next, cytochrome C in mitochondria is released, and finally, the caspase family cascade reaction is activated to induce apoptosis. The caspase family of enzymes involved in apoptosis includes two categories: a. signal initiator (or signal transmission), such as Caspase-9. b. apoptosis effectors, such as Caspase-3. Therefore, we observed the expressions of Bax, Bcl2, cytochrome C, cleaved caspase-9, Caspase-9, cleaved caspase-3, and Caspase-3 related to mitochondrial apoptosis. In the present study, we found that AD LCL cells induced the destruction of mitochondrial membrane potential and increased the expression of cytochrome c and the ratios of cleaved caspase-9/Caspase-9, cleaved caspase-3/ Caspase-3 in co-cultured SKNMC cells. The increase was reversed by catalpol, which was almost the same as NAC. These results indicated that late-onset AD peripheral B cells induced neuronal apoptosis through activating mitochondria-dependent apoptosis pathways. Catalpol alleviated neuronal apoptosis induced by late-onset AD peripheral B cells through inhibiting mitochondria-dependent apoptosis pathways. The further extension of the study will focus on probing whether catalpol improves programmed cell death through other pathways, for instance, ferroptosis, autophagy, etc.

Encouraged by the anti-oxidant and anti-apoptotic effects of catalpol on co-cultured SKNMC cells, all these changes in AD LCL cells induced SKNMCs were reversed by catalpol and NAC, an antioxidant reagent. These results indicated that catalpol could dampen oxidative stress induced by cultured AD LCL cells, so we proceeded to investigate the inherent mechanism of catalpol’s antioxidant function in AD LCL cells-stimulated SKNMCs. There are a series of signaling pathways associated with redox reactions. Among them, the Keap1-Nrf2/ARE signaling pathway is a powerful oxidation-reduction system comprising three components: Keap1, Nrf2, and ARE cis-acting element(45). Keap1 is bound to Nrf2 without oxidative stimulation, making Nrf2 ubiquitinate and degrade (46). Under oxidative stress, the conformation of Keap1 is changed. And thereby, Nrf2 is released, activated, and massively accumulated. Meanwhile, Nrf2 is transferred to the nucleus and combined with ARE to activate downstream gene transcription, ultimately translating a series of antioxidant proteins, such as CAT, SOD, HO-1, and NQO1. Studies *in ****vitro*** showed that catalpol could increase the levels of Nrf2, HO-1, and NQO1 and decrease the expression of Keap1 protein. In addition, it can also promote nuclear translocation of the Nrf2 in SKNMCs co-cultured with AD LCL cells. All these changes in catalpol-treated SKNMCs co-cultured with AD LCL cells were reversed by ML385, a specific inhibitor of Nrf2. Therefore, the anti-oxidant effect of catalpol in AD LCL cells-induced SKNMCs relies on activating the Keap1-Nrf2/ARE signaling pathway(47). In addition, catalpol can down-regulate ROS and MDA levels of ML385 cells, up-regulate the activities of GSH, CAT, and SOD, and inhibit cell apoptosis. The notable point of this experiment is that there is a trend that the levels of all antioxidant markers in the ML 385 group were slightly higher than those in the AD group. Moreover, there was a statistical difference in the content of CAT between the ML385 group and the AD group. Meanwhile, the levels of ROS and MDA in the ML 385 group were slightly lower than those in the AD group. This indicated that, besides activating the Keap1-Nrf2/ARE signaling pathway, catalpol may alleviate oxidative stress through other targets, like the PI3K/AKT/mTOR signaling pathway. 

## Conclusion

We conclude that late-onset AD peripheral B lymphocytes may induce Aβ_1-42_ generation and oxidative stress, resulting in neurotoxicity in the SKNMC neuroblastoma cell line. Catalpol can retard Aβ_1-42_ generation and mitochondria-dependent apoptosis in SKNMC cells co-cultured with AD LCL cells. Meanwhile, catalpol can protect SKNMC cells against late-onset AD peripheral B cells-induced oxidative stress injury and apoptosis by activating the Keap1-Nrf2/ARE signaling pathway. The findings of this study demonstrated that targeting peripheral B lymphocytes may benefit late-onset AD patients, and catalpol may have the potential to be a natural compound that serves as a therapeutic candidate for late-onset AD. The conclusion needs more *in vivo* research to support it later. 
